# Does breakfast skipping alter the serum lipids of university students?

**DOI:** 10.1186/s40795-024-00970-7

**Published:** 2025-03-07

**Authors:** Shazia Parveen Solangi, Naseem Aslam Channa, Amna Yasin Awan, Muhammad Haneef Mugheri, Zeba Hussain Soomro, Lubna Noorani

**Affiliations:** 1 Department of Biochemistry, Shah Abdul Latif University, Khairpur, Pakistan; 2 Institute of Biochemistry, University of Sindh, Jamshoro, Sindh, 76080 Pakistan; 3 Department of Neurology, Civil Hospital, Karachi, Pakistan; 4 Department of Science and Technical Education, Faculty of Education, University of Sindh, Jamshoro, Pakistan

**Keywords:** Breakfast skipper, Nnon-skipper, Lipids, University students, Total cholesterol, Triglycerides, Low density lipoprotein cholesterol, Very low density lipoprotein cholesterol, Total lipids

## Abstract

**Background:**

Breakfast is the first meal of the day which is very important in student’s health. The present study was undertaken to explore the role of breakfast skipping in altering the serum lipids of university students. In this case-control study, university students from various disciplines at Shah Abdul Latif University, Khairpur, Pakistan were selected from January 2021 to August 2023 after obtaining written informed consent.

**Methods:**

Four hundred fifty university students (450) were selected, among them, 158 were Breakfast Skippers (BS) and 292 were breakfast non-skippers (BNS). Of the total, 256 were males and 194 were females with age range of 18–25 years. All participants were enquired about their soci-demographic characteristics and the intake of breakfast during last 3 months. Blood samples were also collected for the serum lipids analysis on auto analyzer ADVIA 1800 S. p-value < 0.05 was kept as level of significant at 95% confidence interval.

**Results:**

We found 292 (60.9%) BNS and 158 (39.1%) BS university students out of 450 university students. Of these, 49.3% were males and 50.7% were female students. Most of the BS (56.9%) had normal weight and 22–23 were the peak age group of breakfast skippers. We found significant variation among age groups for triglycerides (TG), high density lipoprotein cholesterol (HDL-C) and low density lipoprotein cholesterol (LDL-C) in BS. The total cholesterol (TC), TG, LDL-C, very low density lipoprotein cholesterol (VLDL-C), and total lipids (TL) were significantly increased in the BS in comparison to BNS. Increased levels of TG, VLDL-C and TL were detected in male BS compared to male BNS, whereas, reverse was true for HDL-C. the significant higher levels ofTC, HDL-C, LDL-C and TL were found in female BS in comparison to BNS.

**Conclusion:**

In conclusion, the significantly highest concentrations of TC, TG, LDL-C, VLDL-C, and TL are found in the BS compared to BNS. We are fully aware of the fact that the university student’s age group is not involved in lipids related diseases, because the lipid related diseases are the signature diseases of elderly population. Hence, if university students don’t have any other medical condition directly or indirectly involved in affecting serum lipids, then the breakfast skipping may be responsible for altering the serum lipids of university students.

## Introduction

Breakfast is the most essential meal of the day since it is linked to nutritional intake, augmented minerals, and fiber consumption, which is related to maintaining body mass index (BMI) [1]. Breakfast is so critical that numerous researches have been done relating to various factors like BMI, metabolic syndrome [2], mental stress [3], chronic kidney failure [4], gastrointestinal cancers [5], sleep and cardiovascular diseases [6]. One of the previous studies reported that breakfast skipping is associated with high blood pressure [7], metabolic ailment [2], insulin insensitivity [8] and mortality [9, 10]. Breakfast is essential since fasting deprives the body of most vitamins, carbohydrates, minerals, and other nutrients. Those who start their day with nutritious breakfast appear to be more focused, resolute, and fresh than those who skip breakfast [8].

Breakfast skipping is associated with various health-related risk factors, including above mentioned risks as well as heart-related ailments [11]. Blood glucose level in addition to other factors is altered by breakfast skipping. There is no direct association between missing breakfast and weight gain in young adults. It is possible that missing breakfast can affect blood glucose levels since those who skip breakfast have a greater chance to develop type 2 diabetes mellitus [12]. Several investigations have revealed a link between missing breakfast and reduced insulin sensitivity [8, 11], diminished feelings of fullness, and reduced levels of hormones that indicate satiety causes overweight and even obesity and altered biochemistry, specifically serum lipids [13, 14]. The current study sought to improve graduate students’ health by determining their serum lipids using blood samples, along with BMI measurements. The BMI may affect the serum biochemical parameters, like lipid profile (causes dyslipidemia) in breakfast skippers. Skipping breakfast affects many biochemical parameters of the body in general, and the lipids in particular. Lipids synthesize hormones and absorb vitamins in our body; dyslipidemia may cause hormonal and metabolic disorders [15], which in turn, causes obesity and other disorders. University students at the age of 18 to 25 years remains very active, but due to skipping breakfast they become very lazy and become obese. Hence, the present study aimed to study the role of breakfast skipping in altering serum lipids in university students.

## Materials and methods

The present study was conducted from January 2021 to August 2023. This case control study was carried out with a sample size of 450 by simple random sampling technique. Sample size was calculated 138.3 by Cochran’s and Yamane’s formula (Z² Pq/e² where Z is 95% confidence interval and is equal to 1.96, P is prevalence of breakfast skippers in Sindh which is 10% as reported in Sindh, Pakistan [16], q = (1 – p), and e = 0.05 on 95% confidence interval) The 09% non-response rate was also added to obtain (138.3 + 12.4) 150.7 sample size. Hence, we selected 170 breakfast skipper university students, but only 158 breakfast skippers participated in present study.

The total 450 university students (both breakfast skipper and non-skipper) were selected from various disciplines of bachelor level at Shah Abdul Latif University, Khairpur, Pakistan, comprised of 256 males and 194 females with age range of 18–25 years. All participants were divided into two groups based on breakfast intake. Those who skipped breakfast for minimum 3 months were placed in BS (*n* = 158) and those who did not skip breakfast for minimum 3 months were grouped as BNS (*n* = 292) university students. Students who had skipped their breakfast but had a history of systemic ailments (metabolic disorders, anorexia nervosa, diabetes mellitus or any hormonal abnormalities, lactose intolerance, pregnancy, food allergies, and bulimia nervosa or medical condition that may cause changes in serum lipids) were not included in the research. The relevance of the study was explained to all of the registered participants. Socio-demographic characteristics; included age, gender, body mass index (BMI), and some physical parameters like pulse rate and blood pressure were measured by the healthcare professional hired for the study. The main question was about their breakfast; whether the breakfast was taken regularly or not during last 3 months. The anthropometric measurements of each participant, such as weight and height, were calculated for BMI.

Blood samples from only 200 participants (BS = 68 and BNS = 132) were obtained in fasting condition to analyze serum lipids. Fasting blood samples are taken for two reasons: [1] postprandial triglycerides remained elevated for several hours [1], [2] For the reference values majority of plasma lipid concentrations are measured during fasting. Serum lipids were measured by performing various tests, including TC, HDL-C, LDL-C, VLDL-C, TG and TL. The lipid analysis was carried out through an Auto analyzer Siemens ADVIA 1800, (United States) at Hospital of Pir Abdul Qadir Shah Jeelani Institute of Medical Sciences, Gambat, Khairpur Mirs, Pakistan. For the statistical analysis, SPSS Version 22 (SPSS Inc. Chicago, IL) was used to compare the average values of serum lipids between BS and BNS participants, age-wise and gender-wise comparisons were also carried out by z-test and ANOVA (single factor). The level of significance was kept at *p* < 0.05 with 95% confidence interval.

## Results

From a random selection of university students the total number of BS university students was 158, and 292 breakfast non-skippers, including 49.3% males and 50.7% females, were selected for the present study. The majority of BS had normal weight, followed by underweight (20.2%). The highest number of participants were with an age range of 22–23 years (41.1%) in BS compared to BNS university students (Table 1). The number female and male BS university students was almost similar with 50.6% and 49.4% respectively.

We found significant variation among age groups for TG (*p* = 0.038), HDL-C and TL (Table 2). Significantly increased TG (*p* = 0.038) in 24–25 years age group in comparison to other age groups (Table 3). Significantly low HDL-C (*p* = 0.005) was detected in 22–23 years age group, whereas, reverse was true for TL (*p* = 0.0007) in age group 24–25 years when compared to other age groups of BS university sudents (Table 3). At the same time, the significant higher levels of total cholesterol (*p* = 0.00), HDL-C (*p* = 0.003), LDL-C (*p* = 0.024) and TL (*p* = 0.0006) were found in female BS in comparison to female BNS university students (Table 4). Significantly increased levels of VLDL-C (*p* = 0.0003) and TL (*p* = 0.046) were detected in breakfast-skipping male compared to male non-skipper university students. We also found HDL-C (*p* = 0.00) was significantly decreased in male BS university students in comparison to the same group of non-skipping male university students (Table 4).

**Table 1:** Demographic characteristics of breakfast skippers and non-skippers university students

Socio-demographic Characteristics	Breakfast skippers *n* = 158 (%)	Breakfast non skippers *n* = 292 (%)
Gender wise		
Male	78 (49.3)	178 (60.9)
Female	80 (50.7)	114 (39.1)
**Age groups (years)**		
18–19	18 (11.4)	35 (12.0)
20–21	63 (39.9)	130 (44.5)
22–23	65 (41.1)	99 (33.9)
24–25	12 (7.6)	28 (9.6)
Body Mass Index (kg/m^2^)		
Under weight (< 18.5)	32 (20.3)	70 (24.0)
Normal weight (18.5–24.9)	90 (57.0)	160 (54.8)
Overweight (25-29.9)	27 (17.1)	54 (18.5)
Obese (> 40)	09 (5.6)	08 (2.7)
**Blood Pressure (mmHg)**		
Normal	133 (84.2)	273 (93.5)
High blood pressure	05 (3.2)	09 (3.1)
Low blood pressure	20 (12.6)	10 (3.4)
**Pulse rate (beats per minute)**		
Normal	118 (74.7)	260 (89.0)
Abnormal	40 (25.3)	32 (11.0)

**Table 2:** Comparison of serum lipid profile between breakfast skipper and non-skipper university students

Groups	BS (*n* = 68)	BNS (*n* = 132)
TC (< 200 mg/dL)	144.68 ± 35.49*	170.39 ± 42.41
TG (< 150 mg/dL)	136.43 ± 66.63*	178.80 ± 69.06
HDL- C (> 40 mg/dL)	42.10 ± 10.23	45.74 ± 13.12
LDL-C (< 100 mg/dL)	92.11 ± 38.95*	112.46 ± 40.11
VLDL-C (< 30 mg/dL)	27.19 ± 13.13*	36.17 ± 15.06
TL (450–1000 mg/dL)	527.16 ± 125.19*	627.89 ± 144.99

*p (< 0.05) when compared with breakfast non-skippers, BS = Breakfast skipper, BNS = Breakfast non-skipper, TC = Total cholesterol, TG = Triglycerides, HDL-C = High-density lipoprotein cholesterol, LDL-C = Low-density lipoprotein cholesterol, VLDL-C = Very low-density lipoprotein, TL = Total lipids

**Table 3:** Age-wise comparison of serum lipids in breakfast skipper university students

Groups	18–19 Years (*n* = 12)	20–21 Years (*n* = 35)	22–23 Years (*n* = 15)	24–25 Years (*n* = 6)
TC (< 200 mg/dL)	143.3 ± 38.45	126.67 ± 18.98	144.50 ± 39.39	164.50 ± 47.02
TG (< 150 mg/dL)	99.75 ± 18.05	137.88 ± 70.71	113.73 ± 44.41	142.33 ± 45.69^α^
HDL- C (> 40 mg/dL)	43.65 ± 14.26	46.62 ± 13.88	50.88 ± 15.54^α^	41.80 ± 07.37
LDL-C (< 100 mg/dL)	83.41 ± 27.41	93.90 ± 25.15	85.03 ± 43.46	112.50 ± 51.64
VLDL-C (< 30 mg/dL)	32.67 ± 24.87	27.43 ± 13.95	24.20 ± 10.34	28.67 ± 08.96
TL (450–1000 mg/dL)	551.67 ± 207.77	544.01 ± 128.56^α^	532.96 ± 117.39	537.53 ± 56.92

^α^ P(< 0.05) compare among age groups, BS = Breakfast skipper, BNS = Breakfast non-skipper, TC = Total cholesterol, TG = Triglycerides, HDL-C = High-density lipoprotein cholesterol, LDL-C = Low-density lipoprotein cholesterol, VLDL-C = Very low-density lipoprotein, TL = Total lipids

**Table 4:** Gender-wise comparison of serum lipids in breakfast skipper university students

Groups	Male BS (*n* = 32)	Male BNS (*N* = 66)	Female BS (*n* = 36)	Female BNS (*n* = 66)
TC (< 200 mg/dL)	166.56 ± 43.47	160.32 ± 41.00	187.08 ± 37.09*	149.63 ± 27.22
TG (< 150 mg/dL)	183.28 ± 73.19	176.19 ± 74.36	170.11 ± 96.97*	124.91 ± 54.58
HDL- C (> 40 mg/dL)	36.87 ± 10.93*^#^	37.29 ± 10.92	46.96 ± 13.77*	51.61 ± 18.65
LDL-C (< 100 mg/dL)	106.95 ± 39.09	106.25 ± 37.3	136.93 ± 31.77*	107.52 ± 33.95
VLDL-C (< 30 mg/dL)	38.13 ± 15.76*	35.90 ± 15.49	27.42 ± 10.44	26.03 ± 10.97
TL (450–1000 mg/dL)	623.67 ± 159.66*	571.06 ± 113.16	660.39 ± 108.52*	565.48 ± 119.58

*p (< 0.05) when compared with breakfast non-skippers.# p (< 0.05) when compared between males and females, BS = Breakfast skipper, BNS = Breakfast non-skipper, TC = Total cholesterol, TG = Triglycerides, HDL-C = High-density lipoprotein cholesterol, LDL-C = Low-density lipoprotein cholesterol, VLDL-C = Very low-density lipoprotein, TL = Total lipids

**Fig. 1:**
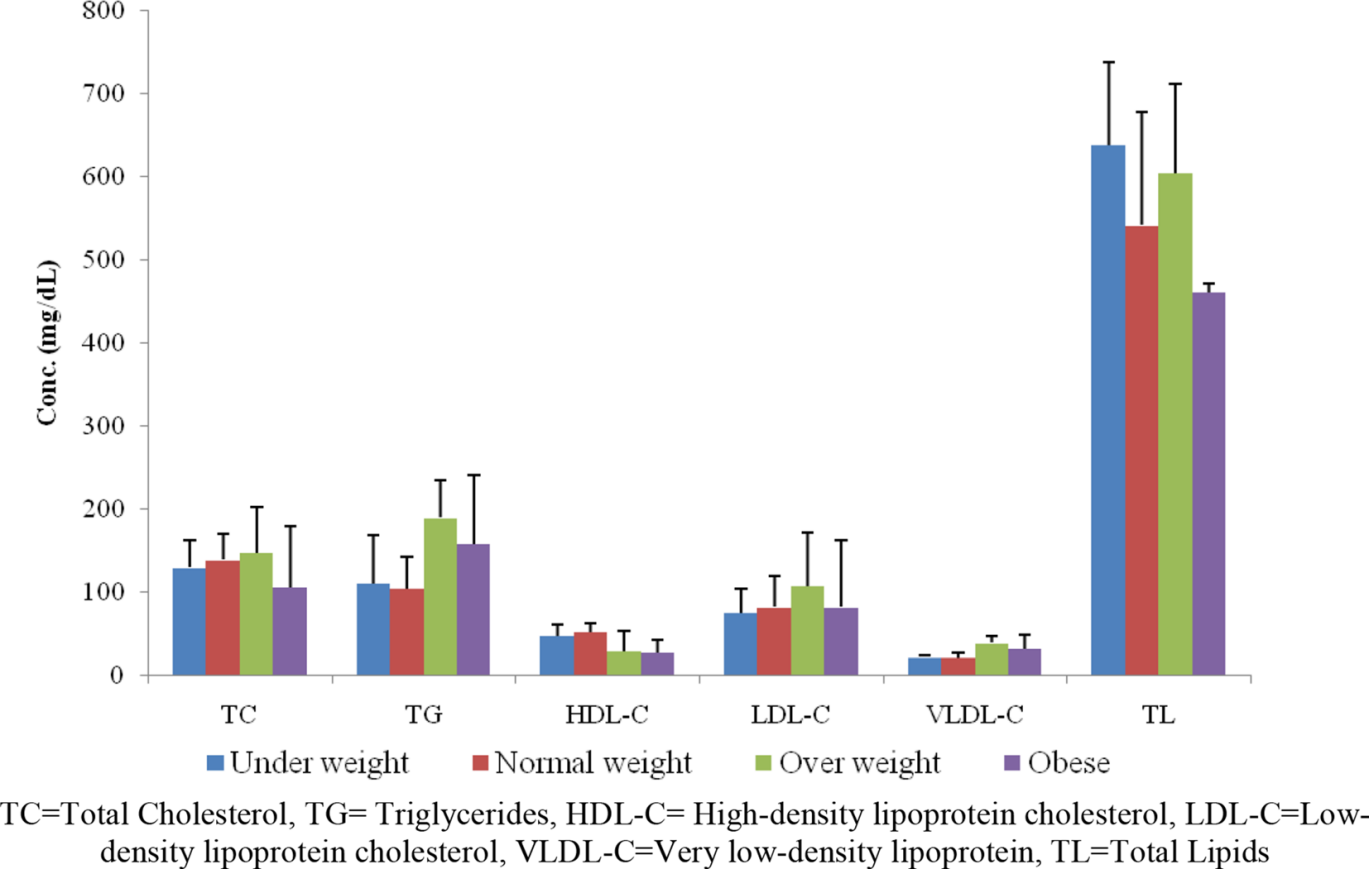
Comparison of serum lipids in breakfast skipper university students with different groups of body mass index. TC = Total Cholesterol, TG = Triglycerides, HDL-C = High-density lipoprotein cholesterol, LDL-C = Low-density lipoprotein cholesterol, VLDL-C = Very low-density lipoprotein, TL = Total Lipids

When the serum lipid profile was compared among different BMI categories of BS university students we found non-significantly increased serum triglycerides, total cholesterol, LDL-C and VLDL-C in overweight as compared to other BMI Categories of BS university students (Fig. 1). We also compared the serum lipid profile among different BMI categories of BNS university students but we didn’t find any significant variation just like BS university students.

## Discussion

It is well known that females frequently skip breakfast compared to males [17]. We also found that more female university students skip breakfast, as shown in Table 1. Young age such as 23 years, is an active age of life in which a person especially female needs proper nutrition to carry out his or her routine activities [18] and skipping breakfast causes malnutrition in that age [19]. It is also proven that skipping breakfast causes metabolic syndrome [20].

The critical inference of the present study is linked to altered serum lipids and BMI (Table 2; Fig. 1), along with the alterations of serum lipids in male BS and female BS, as shown in Table 4. Serum lipids are regulated through complex biological reactions, and Insulin-like growth factor-1 (IGF-1) might play a role as an independent modulator of lipid metabolism. [21]. There are two main categories of cholesterol LDL-C and HDL-C [22]. One of the previous studies supported our results, that a higher level of cholesterol in females with reduced physical activity was noted [18]. Breakfast skipping results in augmented appetite later in the day which leads to the consumption of evening snacks rich in carbohydrates and fats [18], and imbalance the serum lipids, which agrees with the present study. Furthermore it leads to stress as well because chronic stress id related to evening eating choices and overall empty calories in the diet of breakfast skippers, whereas breakfast eaters’ dietary intake is not appear to be affected by chronic stress [23].

From all age groups, the highest serum TG, VLDL-C and TL levels were obtained in the BS university students of 20 to 25 years (Table 3). Since HDL-C has higher protein concentrations and lower in bad fats like LDL-C and VLDL-C, it is thought to benefit the cardiovascular system [24, 18]. Higher levels of TG coupled with HDL-C or LDL-C cholesterols promote atherosclerosis, increasing the amount of fat deposited in artery walls and raising the risk of cardiovascular illnesses [19]. A subtype of LDL-C, usually referred to as bad cholesterol, is VLDL-C. More significant risks of cardiovascular problems are associated with higher levels of VLDL-C and LDL-C [25]. HDL-C functions in processes such as reverse cholesterol transport and inhibits LDL-C oxidation [25], this mechanism of HDL-C is helpful in understanding the imbalance of serum lipids in present study. A study is in contradiction reported that regular breakfast consumption is significantly associated with lower body fatness and healthier dietary habits, although regular breakfast consumption reduces fat in body (depending upon the type of diet as well) [26]. Our results in Table 4 are comparable with other studies, in which they have reported significantly higher fasting triglycerides, VLDL-C, TL and lower HDL-C among male and female BS as compared to BNS [27]. It may also be suggested that certain medical conditions which are directly or indirectly involved in affecting the serum lipids must be excluded for both groups.

In the present study, BMI results showed non-significant differences among different BMI categories (Fig. 1). Previously, it has been reported that increased BMI in BS university students cause reduced LDL-C and total cholesterol [28]. But in contrast present study suggested higher BMI and higher LDL-C and total cholesterol in BS university students. These days, skipping breakfast is becoming common in different nations, including Pakistan, leading to various complications [29, 30]. The development of obesity is highly correlated with behavioral factors, especially inactivity and food consumption. Breakfast, in particular, is a meal that should never be skipped. This practice has been linked to the emergence of obesity or overweight [31], as revealed by the present study (Fig. 1).

## Conclusion

It is concluded that the current study offers the fundamental knowledge of changed serum lipids in breakfast skipper compared to non-skippers. The concentrations of total cholesterol, triglycerides, low density lipoprotein cholesterol, very low density lipoprotein cholesterol, and total lipids are significantly increased in the breakfast skipper compared to breakfast non-skipper university students. Hence, the breakfast skipping may be responsible for altering the serum lipids of university students if they don’t have any other medical condition which may directly or indirectly involved in altering serum lipids. It will help to formulate methods for preventing diseases connected to altered serum lipids. Nevertheless, further studies with a similar design to the present work are necessary to evaluate whether these studies effectively reduce the prevalence of skipping breakfast in university students and/or increase healthy life style in that age group. Furthermore, it is recommended that seminars and workshops be held to raise awareness about the value of breakfast among university students and the school age in spite of everything, the habits start from childhood.

### Limitations

The study is only on university students and the age groups are also very limited. We are well known with the fact that the university student’s age group is not involved in lipids related diseases, because the lipid related diseases are the signature diseases of elderly population. The conclusions were drawn on bachelor level of university students might be affected if university students of masters and doctorate were included, but due to unavailability of funds and facilities, we couldn’t expand the present study. Although, it is very difficult to blame breakfast skipping as the major risk factor for disturbed serum lipids, but if we exclude all factors which are known as a lipid elevators, then the breakfast skipping will be the leading cause of disturbed serum lipids. Unfortunately, we couldn’t exclude all the lipid related diseases except the heart and liver diseases, which are directly related to serum lipid levels. Furthermore, the study on the general population may reveal a significant effect on serum lipids due to skipping breakfast. Nutritional assessment of breakfast skippers is very important to indicate which is also a limitation of the present study.

## Data Availability

The data is in custody of corresponding author and will be provided on demand.
